# DNA methylation profiling to assess pathogenicity of *BRCA1* unclassified variants in breast cancer

**DOI:** 10.1080/15592294.2015.1111504

**Published:** 2016-01-04

**Authors:** Kirsty J Flower, Natalie S Shenker, Mona El-Bahrawy, David E Goldgar, Michael T Parsons, KConFab Investigators, Affect study group, Amanda B Spurdle, Joanna R Morris, Robert Brown, James M Flanagan

**Affiliations:** 1 Epigenetics Unit; Department of Surgery and Cancer; Imperial College London; UK; 2 Department of Histopathology; Hammersmith Hospital; Imperial College London; UK; 3 Huntsman Cancer Institute; University of Utah; Salt Lake City, UT, USA; 4 QIMR Berghofer Medical Research Institute; Brisbane, QLD, Australia; 5 Peter MacCallum Cancer Center; Melbourne, VIC, Australia; 6 Genome Stability Unit; School of Cancer Sciences; University of Birmingham; UK; 7 Section of Molecular Pathology; Institute for Cancer Research; Sutton, UK

**Keywords:** BRCA1, breast cancer, epigenetic, methylation, variants

## Abstract

Germline pathogenic mutations in *BRCA1* increase risk of developing breast cancer. Screening for mutations in *BRCA1* frequently identifies sequence variants of unknown pathogenicity and recent work has aimed to develop methods for determining pathogenicity. We previously observed that tumor DNA methylation can differentiate *BRCA1*-mutated from *BRCA1*-wild type tumors. We hypothesized that we could predict pathogenicity of variants based on DNA methylation profiles of tumors that had arisen in carriers of unclassified variants. We selected 150 FFPE breast tumor DNA samples [47 *BRCA1* pathogenic mutation carriers, 65 BRCAx (*BRCA1*-wild type), 38 *BRCA1* test variants] and analyzed a subset (n=54) using the Illumina 450K methylation platform, using the remaining samples for bisulphite pyrosequencing validation. Three validated markers (*BACH2, C8orf31,* and *LOC654342*) were combined with sequence bioinformatics in a model to predict pathogenicity of 27 variants (independent test set).  Predictions were compared with standard multifactorial likelihood analysis. Prediction was consistent for c.5194-12G>A (IVS 19-12 G>A) (*P*>0.99); 13 variants were considered not pathogenic or likely not pathogenic using both approaches. We conclude that tumor DNA methylation data alone has potential to be used in prediction of *BRCA1* variant pathogenicity but is not independent of estrogen receptor status and grade, which are used in current multifactorial models to predict pathogenicity.

## Introduction

Breast cancer remains the most diagnosed cancer in women, with an overall incidence in the UK of 1 in 8 (http://www.cancerresearchuk.org/cancer-info/cancerstats/). Fortunately, advances in treatment and screening have resulted in reduced mortality rate.^1^ We now understand that breast cancer is a heterogeneous disease, with different individuals benefiting from different treatments depending on hormone receptor expression and genetic mutations. Distinct novel molecular subgroups have been identified using a wide array of technologies, suggesting the need for novel therapies targeting specific molecular alterations.^2,3^ A minority of breast cancers (5-10%) is considered hereditary, and approximately a quarter of these are due to germline mutations in known cancer susceptibilities genes, including *BRCA1* (OMIM# 113705) and *BRCA2* (OMIM# 600185).^4^ Families presenting with multiple breast cancer cases and families with early onset ovarian cancer are currently tested by sequencing these two genes, and this informs clinicians in the management of individual treatment, and decisions concerning screening, chemoprevention or prophylactic surgery for unaffected mutation carriers. Both triple negative tumors and *BRCA1* germline mutated tumors are significantly enriched in the basal subtype, which is associated with poor prognosis. Current estimates suggest that up to 15% of triple negative tumors are *BRCA1* germline mutation carriers.^5,6^

*BRCA1* variants that are considered pathogenic mutations include those leading to protein truncation or mRNA degradation, and risk-associated missense alterations affecting protein function accompanied by clinical evidence of pathogenicity. However, there are also a considerable number of *BRCA1* variants of uncertain clinical significance (>500 different variants^7^) namely missense variants with undetermined effect on function or risk, small insertions or deletions, or alterations in noncoding sequence. Attempts to classify these unclassified variants have utilized a range of analyses. Segregation data can be used to identify if a variant tracks with disease within a family; however, this is difficult to apply to some variants since they are individually rare.^8^ *In silico* approaches have been used to examine sequence evolutionary conservation predicting the effect of specific amino acid substitutions^9^ and potential splicing alterations.^10^ A multifactorial model has been developed to integrate these different data together, starting with an empirical prior probability based on bioinformatics predictions and incorporating likelihood ratios derived from independent data sources to generate a posterior probability of pathogenicity.^8^ The advantages of using this approach are that it incorporates all available information from multiple data types in a single analysis with a numeric output and allows for the addition of extra data.^11^ The posterior probability generated can then be used to classify the variant into 1 of 5 classes: Class 1 (probability <0.001) – non pathogenic, or of no clinical significance; Class 2 (probability 0.001-0.049) – likely non pathogenic or of little clinical significance; Class 3 (probability 0.05-0.949) – uncertain; Class 4 (probability 0.95-0.99) – likely pathogenic; Class 5 (probability >0.99) – definitely pathogenic.^12^

We have previously shown that breast tumors from patients with pathogenic germline *BRCA1* mutations have distinct DNA methylation profiles compared to familial breast cancer cases with no *BRCA1* or *BRCA2* mutations (BRCAx).^13^ In this previous study, 81.3% of tumors with germline mutated *BRCA1* were correctly predicted using a support vector learning machine approach based on DNA methylation data. The methylation profiles of BRCAx tumors are very similar to those of sporadic breast cancers.^14^ The present study aimed to assess the DNA methylation of both published candidate genes and novel regions that differ between tumors from germline *BRCA1*-mutation carriers (referred to as *BRCA1* tumors) and tumors from BRCAx families (germline *BRCA1*-wild type), with the goal of providing an additional tool useful for the classification of unclassified *BRCA1* variants.

## Results

### DNA methylation of prior candidate genes is dependent on estrogen receptor (ER) status

Candidate regions were identified from previous data^13,14^ in which DNA methylation levels were significantly different between tumors of *BRCA1*-mutation carriers and those of BRCAx individuals (**Table S1**). Twelve candidate regions were analyzed in bisulphite converted DNA from 150 tumors (*BRCA1*, BRCAx and *BRCA1* test variant) by pyrosequencing.  We validated the expected difference between *BRCA1* and BRCAx tumors for 6/12 candidate regions, observing lower median methylation in *BRCA1* tumors for five candidate regions [*CD9* (OMIM# 143030), *SGK1* (OMIM# 602958), *ERCC3* (OMIM# 133510), and 2 regions in *FGF2* (OMIM# 134920), wilcoxon rank sum test, *P*<0.05] and significantly higher methylation in *BRCA1* tumors for one gene [*CD40* (OMIM# 109535), *P*=0.02] (**Table S1**). Logistic regression analysis showed that methylation status was significantly associated with *BRCA1* mutation status (**Table S1**). Since ER status is a known predictor of *BRCA1* mutation status [local recurrence (LR) ranging from 0.08-0.90 for ER-negative status, dependent on grade],^15^ we assessed whether differences observed in methylation between *BRCA1* and BRCAx tumors in the remaining five candidate regions of interest were independent of these known histopathological predictors. Using a generalized linear model we showed that methylation differences observed in these five regions were associated with ER status (*P*-value <0.05) (**Table S1**). ER status and mutation status are highly associated in this dataset [generalized linear model (glm) P-value = 4.31 × 10^−7^], and none of the five candidates were significantly independent from ER status, indicating that they should not be used in conjunction with existing pathology LRs^15^ in multifactorial modeling for prediction of variant pathogenicity.  In light of these findings, further analyses considering grade, another predictor of mutation status, were not pursued.

### Genome-wide analysis identifies novel differentially methylated loci that define *BRCA1* mutation status

**Figure 1.**
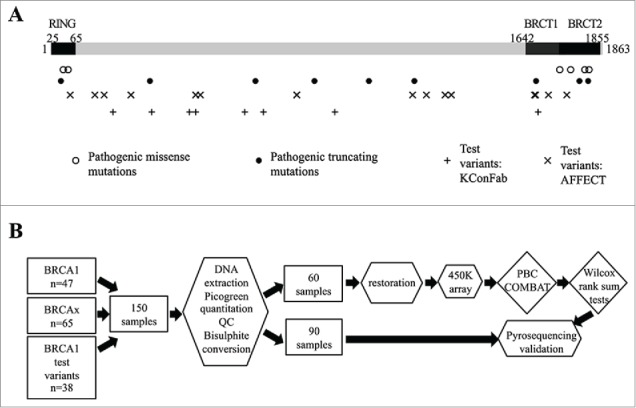
**Study Design. (A)** Representation of the exonic position of the known mutations in the *BRCA1* gene used in this study, including pathogenic (missense and truncating) and test variants. Some variants are represented by multiple tumors. **(B)** Flow diagram illustrating study design. Rectangular boxes indicate samples, hexagonal boxes represent experimental processes, and diamond-shaped boxes denote bioinformatics processes or analysis.

**Figure 2.**
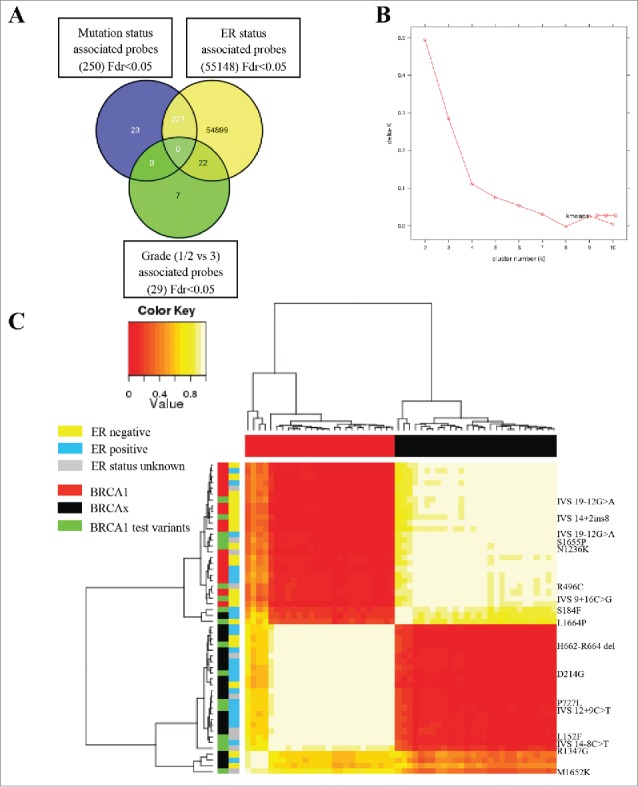
**Methylation array (450K) analysis defines pathogenic and neutral variants. (A)** Venn diagram representing the comparison of significant probe lists (FDR <0.05), showing 23 probes only significant in the mutation status analysis. **(B)** Consensus clustering using 18 probes with a difference in methylation between *BRCA1* mutated and BRCAx greater than 5% identified two main clusters, as shown by kmeans plot. **(C)** The resulting correlation matrix from these two clusters shows that these clusters correlate with mutation status. Unclassified variants in green clustered with either the *BRCA1* tumors or the BRCAx tumors.

The overall study design is shown in **Figure. 1A-B**. The Illumina HumanMetylation 450 (450K) BeadChip array has successfully been used to assess DNA methylation in formalin-fixed, paraffin-embedded (FFPE)-derived DNA samples using appropriate quality control and restoration methods.^16^ We selected 60 samples [*BRCA1* pathogenic variants (n=20), BRCAx (n=20), and *BRCA1* test variant (n=20)] for array analysis, of which 54 (90%) samples passed quality control [*BRCA1* pathogenic (18/20), BRCAx (19/20), *BRCA1* test variant (17/20)]; 482351/485577 (99.3%) probes passed quality control (QC). The Wilcoxon rank sum test was used in three analyses to identify (i) probes with significant differences between tumors from patients with germline *BRCA1* mutations and tumors from BRCAx cases [false discovery rate (FDR) q<0.05, n=250 probes]; (ii) probes with significant differences between ER positive tumors and ER negative tumors (FDR q<0.05, n=55148 probes); and (iii) probes with significant differences between low grade (Grades 1 and 2) and high grade (Grade 3)  tumors (FDR q<0.05, n=29 probes). Using a Venn diagram we show that 23 probes were apparently unique to the mutation class analysis (**Fig. 2A**). Probes with an absolute methylation difference between groups of less than 5% were excluded, leaving 18 probes with a range of absolute difference in methylation between 5% and 30%. We used consensus clustering of the array data using the 18 selected probes that differentiated *BRCA1* pathogenic tumors from the BRCAx tumors to determine with which cluster each of the *BRCA1* test variant tumors clustered. This method confirmed only two tumor clusters based on these data (**Fig. 2B**) and found nine *BRCA1* test set variant tumors clustered with pathogenic *BRCA1* mutated tumors, and eight *BRCA1* test set variant tumors clustered with BRCAx tumors.(**Fig. 2C**)

Pyrosequencing assays were designed for these 18 loci, and optimized for 16. Methylation analysis of these regions was conducted for 150 FFPE samples (54 matched to the array, the 90 independent samples, and the 6 that did not pass array QC criteria). Intraclass correlation coefficients (ICC) of assays were calculated to compare the β values obtained from the array to pyrosequencing values for matched samples, and the Wilcoxon rank sum test used to validate the difference between the *BRCA1* mutated and BRCAx group in the independent samples (**Table 1**). A significant difference (*P*<0.05) between *BRCA1* samples and BRCAx samples was validated for 5 loci [450K probe IDs cg24667115 (*BACH2*  OMIM# 605394), cg03029255 (*C8orf31*), cg02502358 (*LYRM9*), cg21645762 (*LOC654342*) and cg12472473 (chr13q34)] in the independent group of samples.

**Table 1.** Candidate gene validation by pyrosequencing.

		Array data (β values)							Samples Passed QC				Pyrosequencing Validation (% methylation)				
Region Name	Probe ID	Wilcox mut q value FDR^*^	wilcox ER q value FDR^#^	Wilcox grade q value FDR^$^	BRCA1 mutated Median β value	BRCAx Median β value	Methylation difference (β value) [Bx-BRCA1]	SNP in probe targeted CpG?	Total (/96)	BRCA1 (/28)	BRCAx (/47)	Test set (/21)	Wilcox BRCA1 mutated vs. BRCAx *P* value	Median methylation BRCA1 mutated	Median methylation BRCAx	Methylation difference (%) [Bx-BRCA1]	ICC: array β values vs. Pyrosequencing %^†^
BACH2	cg24667115	0.0494	0.0771	0.2378	0.28	0.51	0.23		64	19	27	18	0.0070	35.4	51	15.6	0.717
C8orf31	cg03029255	0.0462	0.1118	0.1711	0.71	0.41	-0.3		47	16	11	20	0.0015	73.6	26.2	-47.4	0.715
C17orf108	cg02502358	0.0494	0.0571	0.4768	0.16	0.07	-0.09		59	19	29	11	0.0454	49.6	10.4	-39.2	0.666
LOC654342	cg21645762	0.0494	0.0542	0.2759	0.77	0.61	-0.16		34	9	18	7	0.00003	88.1	73.8	-14.3	0.642
ch13q34	cg12472473	0.0350	0.7011	0.5566	0.91	0.75	-0.16	Y	53	19	25	9	0.0428	66.1	31.9	-34.2	0.477
chr1p34.2	cg07405182	0.0462	0.2613	0.2437	0.87	0.59	-0.28		48	11	27	10	0.1791	45	62	17	0.469
chr15q22.31	cg08036487	0.0462	0.0542	0.2759	0.75	0.56	-0.19		42	11	25	6	1.0000	52.5	51	-1.5	0.431
chr3q25.2	cg02071853	0.0462	0.0633	0.5381	0.72	0.51	-0.21		36	10	19	7	0.1644	84	71.2	-12.8	0.360
ZNF319	cg06866605	0.0494	0.0771	0.2696	0.9	0.94	0.04		53	12	31	10	0.2972	81.1	83.8	2.7	0.260
chr6q27	cg26074411	0.0494	0.1382	0.1243	0.9	0.85	-0.05		33	6	19	8	0.3319	98.8	88.6	-10.2	0.236
INTS1	cg11644627	0.0490	0.0810	0.8266	0.81	0.89	0.08		52	15	28	9	0.9498	76.4	71.6	-4.8	0.111
chr8p23.1	cg04039177	0.0228	0.2284	0.7353	0.57	0.41	-0.16	Y	45	12	25	8	0.4134	74	68	-6	0.164
DLC1	cg00933411	0.0494	0.0849	0.7508	0.37	0.23	-0.14		33	9	20	4	0.1272	46.6	28.3	-18.3	0.119
TJAP1	cg16759204	0.0358	0.1441	0.1885	0.7	0.57	-0.13		51	13	29	9	0.5013	80.6	87.9	7.3	0.111
PLAGL1	cg02697107	0.0494	0.6577	1.0000	0.86	0.81	-0.05		52	15	28	9	0.7529	88.1	84.1	-4.0	0.103
CRTAC1	cg23801028	0.0437	0.1168	0.4680	0.47	0.63	0.16		66	19	34	13	0.6278	71.7	65.9	-5.8	0.033
chr16q24.1	cg01385438	0.0448	0.0602	0.3067	0.9	0.83	-0.07		X	x	x	x	ND	ND	ND	ND	ND
KRT6C	cg09440385	0.0494	0.0734	0.5752	0.96	0.91	-0.05		X	x	x	x	ND	ND	ND	ND	ND

* False Discovery Rate corrected *P* value for Wilcoxon rank sum test: BRCA1 mutated tumors vs. BRCAX tumors, β values, array samples

# False Discovery Rate corrected *P* value for Wilcoxon rank sum test: ER positive tumors vs. ER negative tumors, β values, array samples

$ False Discovery Rate corrected *P* value for Wilcoxon rank sum test: grade 1+2 vs. grade 3, β values, array samples

† Intraclass Correlation Coefficient (ICC) calculated using array β values and pyrosequencing methylation percentages for the same sample (n between 26 and 54, dependent on assay)

ND Not Done - assay design was not possible/could not be optimized

**Figure 3.**
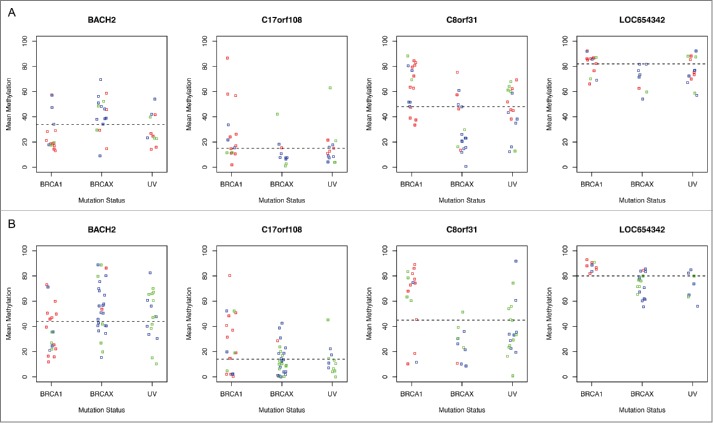
**Validation of** ***LYRM9*****,** ***BACH2*****,** ***LOC654342,*** **and** ***C8orf31*** **loci. (A)** Strip charts show array β values for the four validated loci plotted by mutation status. Blue dotted line is plotted at the median of *BRCA1* and BRCAx samples. ER negative samples are colored red, ER positive samples are colored blue, and those samples for which ER status is unknown are colored green [*BRCA1* (n=18), BRCAx (n=19), *BRCA1* test variant (n=17), except *BACH2,* where *BRCA1* n=17]. **(B)** Strip charts showing the pyrosequencing methylation value of the independent group of samples validating the difference observed on the array [*LYRM9*:*BRCA1* (n=19), BRCAx (n=29), test variant (UV) (n=11); *BACH2*:*BRCA1* (n=19), BRCAx (n=27), test variant (UV) (n=18); *C8orf31*: *BRCA1* (n=16), BRCAx (n=11), test variant (UV)(n=20). *LOC654342*:*BRCA1* (n=9), BRCAx (n=18), test variant (UV) (n=7)].

However, the cg12472473 (13q34) locus harbored a CG>TG SNP with a minor allele frequency (MAF) of 0.2 within the CpG dinucleotide of interest, and pyrosequencing genotyping showed that the T genotype was overrepresented in the BRCAx group (chi squared test, *P*<0.05). This region was therefore excluded from further analysis. The technical and biological validation of the four loci is shown in **Figure 3**.  Further, despite selecting array probes that apparently predicted mutation status independent of ER and grade status (see **Fig. 2**), logistic regression analysis of the pyrosequencing data showed that probes cg24667115 (*BACH2*) and cg02502358 (*LYRM9*) were not independently associated with mutation status when ER status was also included in the model (**Table 2**). Additionally, there was evidence that methylation status of cg02502358 (*LYRM9*), cg24667115 (*BACH2*) and cg21645762 (*LOC654342*) were correlated with grade, although this analysis was based on approximately half of the individuals, for which we had information on all variables, which reduces the statistical power of the analysis.

**Table 2.** Logistic regression analysis of candidate genes.

Assay	Logistic Regression *P* values						Number of samples included in logistic regression^*^
	mut~meth	mut~meth^+^ER		mut~meth^+^ER+grade			
		Meth	ER	meth	ER	grade	
BACH2	0.0265	0.0607	0.0002	0.0848	0.0131	0.0976	61
C8orf31	0.0003	0.0063	0.0101	0.0131	0.0866	0.3721	48
C17orf108	0.0166	0.1010	0.0002	0.1145	0.0495	0.0146	49
LOC654342	0.0038	0.0301	0.0043	0.0829	0.0645	0.0516	37

* only samples with complete data (methylation, ER status, and grade) were used in these logistic regressions

As for candidate region analysis described above, these findings suggest that the majority of methylation probes that are associated with mutation status are, in fact, not independent of ER or grade.  Thus, with the exception of cg03029255 (*C8orf31*), the markers identified in this study should not be used in conjunction with existing ER and grade LRs in multifactorial likelihood prediction of variant pathogenicity.

### Predictive capacity of different methylation markers of mutation status

Pearson's correlation coefficient was calculated for the pyrosequencing data of each locus against the others, and the R^2^ value for all comparisons was less than 0.2, indicating a low extent of correlation [R^2^ (*BACH2* vs. *C8orf31*) = 0.0436, R^2^ (*BACH2* vs. *LOC654342*) = 0.1719, R^2^ (*BACH2* vs. *LYRM9*) = 0.0207, R^2^ (*C8orf31* vs. *LOC654342*) = 0.1935, R^2^ (*C8orf31* vs. *LYRM9*) = 0.0953, R^2^ (*LOC654342* vs. *LYRM9*) = 0.0356].

The β values at these four loci were converted to z-scores and combined in a logistic regression model. Due to missing data for some of these markers, there were not enough data points to fit a model to more than three markers. *LYRM9* was therefore removed from the model. Using the Leave One Out Cross Validation (LOOCV) method based on methylation data of the three remaining markers alone, 32 out of 37 samples are correctly predicted (86%). The pyrosequencing methylation data was also converted to z-scores, and the combined logistic regression model correctly predicted 78 out of 97 [80% (positive predictive value =76%), (negative predictive value =79%)] of samples with known mutation status using only methylation data.

**Figure 4.**
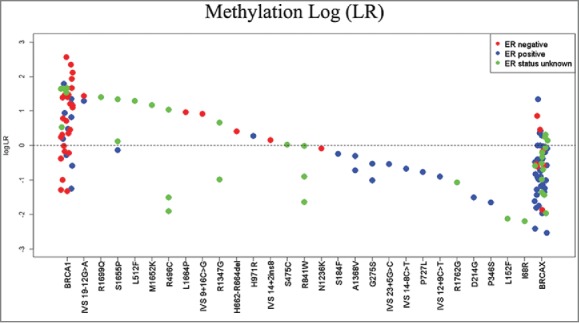
**Combined methylation likelihood ratios.** LRs were calculated for each sample using the model including only methylation. The log of the combined LR is plotted here corresponding to each variant assayed.

The logistic regression model including methylation of the three validated loci was used to predict all tumor samples from test set variant carriers based on methylation alone, and results were compared to current classification based on other evidence including ER and grade among other variables (**Table 3**). To represent these predictions for all samples based on their methylation, included those of known pathogenicity, likelihood ratios (mLRs) were calculated using the probability values generated by the prediction model (probability of pathogenicity divided by 1 minus the probability of pathogenicity, shown in **Tables S2 and S3**), and the log of these values was plotted (**Fig. 4**). This indicates which samples of the same variant were similar in pathogenicity prediction (for example, *BRCA1* IVS 19-12 G>A) and those that are more discordant (Arg1347Gly), and also illustrates the spread of methylation prediction for the known variants (*BRCA1* and BRCAx extremes of plot).

**Table 3.** Combined summary predictions of all unknown variants analyzed.

Number of tumors	Number of independent tumors^*^	HGVS Nuc	HGVS Prot	Prior probability	Current posterior probability^†^	Current Classification^†^	Posterior probability using methylation only	Classification using methylation only
3	3	c.1486C>T	p.Arg496Cys	0.02	0.00089	Class 1	0.00009	Class 1
3	2	c.2521C>T	p.Arg841Trp	0.02	2.29E-12	Class 1	0.0005-0.00006	Class 1
3	3	c.4963T>C	p.Ser1655Pro	0.03	^∞^	Class 3	0.39565	Class 3
2	1	c.4103C>T	p.Ala1368Val	0.02	0.00163	Class 2	0.010-0.004	Class 2
2	1	c.823G>A	p.Gly275Ser	0.02	0.00427	Class 2	0.002-0.006	Class 2
2	2	c.5194-12G>A	IVS	0.34	0.99999	Class 5	0.99638	Class 5
2	2	c.4039A>G	p.Arg1347Gly	0.02	2.04E-12	Class 1	0.00967	Class 2
1	1	c.641A>G	p.Asp214Gly	0.02	0.07426	Class 3	0.00065	Class 1
1	1	c.1984_1992del	p.His662-Arg664del	0.02	0.06058	Class 3	0.04922	Class 2
1	1	c.2912A>G	p.His971Arg	0.02	0.00025	Class 1	0.03682	Class 2
1	1	c.203T>G	p.Ile68Arg	0.66	^∞^	Class 3	0.01215	Class 2
1	1	c.4185+9C>T	IVS	0.02	0.00232	Class 2	0.00258	Class 2
1	1	c.4484+2_4484+3ins8	IVS	0.97	0.98991	Class 4	0.97881	Class 4
1	1	c.4485-8C>T	IVS	0.04	^∞^	Class 3	0.00884	Class 2
1	1	c.5467+5G>C	IVS	0.34	0.19022	Class 3	0.13048	Class 3
1	1	c.593+16C>G	IVS	0.02	^∞^	Class 3	0.14272	Class 3
1	1	c.454C>T	p.Leu152Phe	0.02	^∞^	Class 3	0.00016	Class 1
1	1	c.4991T>C	p.Leu1664Pro	0.03	1.18E-05	Class 1	0.22327	Class 3
1	1	c.1534C>T	p.Leu512Phe	0.02	0.00014	Class 1	0.28730	Class 3
1	1	c.4955T>A	p.Met1652Lys	0.66	^∞^	Class 3	0.96673	Class 4
1	1	c.3708T>G	p.Asn1236Lys	0.02	^∞^	Class 3	0.01645	Class 2
1	1	c.1036C>T	p.Pro346Ser	0.02	0.00427	Class 2	0.00046	Class 1
1	1	c.2180C>T	p.Pro727Leu	0.02	0.00017	Class 1	0.00352	Class 2
1	1	c.5096G>A	p.Arg1699Gln	0.66		Intermediate risk - based on segregation analysis (Spurdle et al., 2015)	0.97998	Class 4
1	1	c.5284A>G	p.Arg1762Gly	0.03	^∞^	Class 3	0.00247	Class 2
1	1	c.551C>T	p.Ser184Phe	0.02	^∞^	Class 3	0.01149	Class 2
1	1	c.1423A>T	p.Ser475Cys	0.02	0.00395	Class 2	0.02111	Class 2

* only tumors from different individuals can be considered independent

† based on segregation analysis alone (Arg1699Gln) or multifactorial likelihood analysis incorporating segregation, pathology, and other data points (see supplementary **Table S2**)

∞ combined LR does not pass thresholds recommended as per ENIGMA *BRCA* classification guidelines (http://www.enigmaconsortium.org/), namely LR of <0.5 (to reach final Class 2 or 1), or >2.0 (to reach final Class 4 or 5) and so should be considered Class 3 (uncertain).

Of the 27 unique variants in the test set, one is known to be an intermediate risk allele from extensive segregation studies (Arg1699Gln),^17^ and 15 have sufficient information available to place them in Class 1, 2, or 4, 5, based on multifactorial likelihood analysis that uses currently accepted predictors of mutation status. Of the latter, one was considered Class 5 (pathogenic), one Class 4 (likely pathogenic), seven were Class 1 (non pathogenic), 6 were Class 2 (likely non pathogenic), and the remainder were Class 3 (uncertain) after classification. Comparing these segregation and multifactorial results to those from methylation analysis alone, the intermediate risk variant Arg1699Gln had an LR of 25.218, the two Class 5 variant IV19-12 G>A samples had LRs of 19.6 and 27.24 in favor of pathogenicity, and the Class 4 variant *BRCA1* IVS14+2 ins8 had an LR of 1.43. The Class 1 variants had LRs ranging from 0.008 to 19.75 (with more than 60% of these <1), while the Class 2 variants had LRs ranging from 0.02 to 1.05.

There were 27 unique *BRCA1* variants within these 37 individuals, five of which had at least two independent tumor samples from different analyzed individuals, and the logistic regression model resulted in probabilities of pathogenicity for each sample. These probabilities were converted to LRs and combined by multiplication. Prior probabilities for these variants based on bioinformatics analysis (see Materials and Methods) were combined with the likelihood ratio calculated for each variant, and these posterior probabilities are summarized in **Table 3**. Using the established posterior probability cut-offs for the IARC 5 tier classification system,^12^ *BRCA1* IVS 19-12 G>A would be classified as pathogenic (Class 5). Arg496Cys and Arg841Trp would be classified as neutral (Class 1).   This would suggest that, at least based on current information, the likelihood ratios derived from the methylation results based on three probes are concordant for the majority of variants with the largest discordance observed being with Class 3 (uncertain) variants. When these likelihood ratios are combined with the bioinformatics prior probabilities, the predictions for Class 1 and 2 variants become far more consistent, but the prediction for *BRCA1* IVS14+2 ins8 becomes more uncertain.

## Discussion

Multiple studies have identified distinct epigenetic profiles that correlate with different breast cancer subtypes and/or ER status. The most recent example is data from The Cancer Genome Atlas (TCGA) project, which used shared probes between 27K and 450K analysis on 802 tumors to distinguish five groups. Two groups were highlighted: Group 3 showed a hypermethylated phenotype and was enriched for luminal subtype ER positive tumors, while Group 5 showed the lowest levels of methylation in the probes selected and this group overlapped with basal-like subtype ER negative tumors.^2^ The association between methylation and subtype/ER status is recapitulated by several other studies.^14,18-24^ which show a lower level of methylation in ER negative/basal-like tumors compared to ER positive/luminal tumors. We previously showed a difference in methylation between familial breast tumors with different *BRCA1* germline mutations compared to BRCAx, and found that the methylation clusters formed were independent of the intrinsic subtype, and therefore had the potential to be independent of ER status.^13^ We hypothesized that this difference in DNA methylation profile could contribute evidence to classify *BRCA1* sequence variants of uncertain clinical significance.

We undertook a two-phase study to assess the value of DNA methylation for predicting pathogenicity of *BRCA1* variants, the strength of which was the large number of *BRCA1* mutant carrier and BRCAx (*BRCA1* wild type) tumors used as a reference to generate the predictive algorithm. There is no evidence to suggest that BRCAx tumors have significantly different methylation profiles to sporadic breast cancers.^14^ The first phase analyzed 12 previously reported candidate regions and validated 5 regions to be strongly associated with mutation status, but also showed that these associations were not independent of ER status or grade. In the second phase, using the Illumina 450K BeadChip array to conduct a genome-wide analysis, we showed that DNA methylation profiles are largely driven by ER status, with 55148 probes significantly associated with ER status. The mechanism by which this occurs is still not understood, and it is unknown whether these changes in methylation occur at ER target genes or as a consequence of ER transcriptional regulation. Very few probes were significantly associated with grade.

Using the Illumina 450K BeadChip array, we also identified 250 loci associated with mutation status, 23 of which initially appeared to be independent of tumor ER status and grade. Of these, 18 had an absolute methylation difference between groups of greater than 5%, and using these 18 novel loci, we were able to cluster the samples into two groups of *BRCA1* carrier and BRCAx tumors. The test set samples from variant carriers clustered with the *BRCA1* pathogenic or BRCAx subgroups.  Of these 18 loci, four sites were validated by pyrosequencing in an independent group of samples, but logistic regression suggested that for all but one probe, the association was not truly independent from ER status and grade in the independent samples. Using the data to predict the mutation status from these methylation values alone, a logistic regression model was created and used to predict the pathogenicity of the 27 different test variants, and these predictions based on methylation data were compared to current evidence regarding pathogenicity using other information sources (summarized in **Table 3**, detailed in **Table S4**). Combining the predictions from the methylation models with known prior probabilities for each variant, *BRCA1* IVS 19-12 G>A would be classified as pathogenic (Class 5), and Arg496Cys and Arg841Trp would be classified as neutral (Class 1). These classifications matched those based on multifactorial likelihood analysis or segregation analysis, so the utilization of this methylation data supports the predictions of these variants.

Further comparison of the current classifications and the methylation derived classifications revealed very few major inconsistencies; a further 8 variants (Ala1368Val, Gly275Ser, Arg1347Gly, His971Arg, *BRCA1* IVS 12+9 C>T, Pro346Ser, Pro727Leu, Ser475Cys) were classified by both methods as Class 1 (non pathogenic) or Class 2 (likely non pathogenic). A further three variants (Ser1655Pro, *BRCA1* IVS 23+5 G>C, *BRCA1* IVS 9+16 C>G) were classified by both methods in the broad uncertain Class 3, indicating for these few variants there is not enough evidence to categorize them confidently by either method. The methylation derived classification correctly predicts the Class 4 variant within this test set, *BRCA1* IVS 14+2 ins 8, as Class 4. Due to missing pathology, lack of previous investigation or a combined LR between 0.5 and 2.0 (which therefore does not pass thresholds recommended by the ENIGMA BRCA classification guidelines, see http://www.enigmaconsortium.org/), six variants do not have a current classification based on multifactorial likelihood analysis or segregation analysis but are all classified by the methylation model as Class 1 or Class 2 (Ile68Arg, Leu152Phe, *BRCA1* IVS 9+16C>G, Asn1236Lys, Arg1762Gly, Ser475Cys). It is therefore difficult to assess the validity of the methylation predictions for these variants. Finally, two variants with current uncertain classification (Asp214Gly and His662-Arg664del) share methylation characteristics with the less pathogenic variants of Class 1 and 2.

The relationship between loss of BRCA1 function and a direct or indirect mechanism that influences DNA methylation in normal or cancerous breast tissue remains unclear and an area for future research. We observed two samples that exhibited *BRCA1* promoter methylation (>10% by pyrosequencing); one BRCAx sample and one test variant sample (*BRCA1* IVS 9+16C>G). The BRCAx sample was predicted to be more similar to a pathogenic *BRCA1* mutation (probability from methylation model = 0.8778, LR=7.18) (**Table S3**), while the test variant also had a probability of 0.89 and LR of 8.1575 (**Table S2**), indicating the potential similarity with pathogenic variants; however, the posterior probability for this sample was 0.14 and remained Class 3 (uncertain). Therefore, it remains unclear whether *BRCA1* promoter methylation, which may be more heterogeneous in the different tumor cells, influences the DNA methylation profile in the same manner as germline *BRCA1* pathogenic mutations.

The *BRCA1* variant Arg1699Gln has been shown to be defective in the formation of foci in response to DNA damage, and also has some effect on transcriptional activity; however, this variant was not categorized as high risk, due to the mixture of intermediate and defective phenotypes in the functional assays^25^ Another study, using clinical parameters to assess pathogenicity, classified this variant as deleterious,^26^ and a structural approach also indicated a pathogenic phenotype.^27^ A recent functional complementation assay.^28^ provided additional evidence of pathogenicity, based on proliferation and cisplatin response assays, as well as sensitivity to PARP inhibitors. The most extensive genetic study of 68 families showed that this variant was associated with an intermediate risk.^17^ Our logistic regression model based on DNA methylation indicated that this variant was probably pathogenic, returning a probability of pathogenicity of 0.97998. The methylation data supports increased risk known to be associated with this variant.

One of the limitations of our study is the small number of ER positive tumors with *BRCA1* pathogenic mutations, and ER negative tumors without any known mutations (BRCAx). However, this is indicative of an existing bias, which makes it difficult to discover mutation specific changes, when the ER status is so closely related to the methylation levels, and this bias is inherent in most available study data. An interesting question to consider is whether the ER positive *BRCA1* tumors are in fact sporadic cases; recent studies, including whole genome massively parallel sequencing analysis of both ER positive and negative *BRCA1* mutated tumors, provide evidence that this is not the case.^29,30^ A further limitation of this study is the assumption, based on previous data,^13^ that all *BRCA1* mutant tumors have similar profiles for different missense or truncating mutations and the underlying hypothesis that aberrant BRCA1 function is the driver of the aberrant methylation profile. Much larger numbers of independent tumors with the same *BRCA1* variants would be required to address this limitation. A limitation of using pyrosequencing is that the fragmented DNA from FFPE samples resulted in higher numbers of samples failing QC in each of the assays. Array based methods have restoration procedures that are unsuitable for pyrosequencing, and thus we found between 30-75% of samples variably failed QC for each assay (**Table 1**). Due to the relative rarity of these variants, FFPE blocks are the most available source of material; however, they are subject to degradation in the quality of DNA, as well as lower yields depending on size of tumor and amount of remaining archival tissue.  Furthermore, we did not have access to adjacent normal tissue of the same patients, which may have provided further information on whether the genetic variants influence the methylation of normal tissue in addition to the tumor tissue. Lastly, common to many tumor methylation profiling studies, we have not accounted for the numerous environmental exposures and factors that might influence or confound the methylation profile of the tumor, such as age, BMI, alcohol, smoking, and the tumor microenvironment. Future studies should record such data on subjects to allow adjusting for these factors in defining differential methylation.

In conclusion, we have developed a methylation-based prediction tool that adds useful information that may be included in the multifactorial model to classify *BRCA1* variants of uncertain clinical significance, but note that most methylation markers identified are not truly independent of ER and grade status. Thus, methylation data should be considered as complementary to other pathology data for multifactorial likelihood prediction modeling and may be useful for classifying variants with tumors where these clinical variables may be absent. Measurement of methylation allows a quantitative approach and, with the appropriate controls, methylation characterization provides promise to reduce inter-laboratory variability in pathological marker calls(such as immunohistochemistry methods^31-33^) and improves application of this alternative tumor pathology characteristic for mutation prediction studies in the future. The methylation data measured using pyrosequencing can be incorporated into prediction models easily, and may capture risk information associated with multiple other pathological features. High-throughput analysis of multiple samples is also feasible, and we have shown here that FFPE-derived DNA is amenable to this analysis, allowing the use of archival tissue, essential for the investigation of rare variants.  In summary, this work suggests that the methylation markers will have value for future variant classification for *BRCA1* and potentially for other genes with known tumor methylation phenotypes, such as *MLH1* (OMIM# 120436) in colon cancer (Cancer Genome Atlas Network, 2012b).

## Materials and Methods

### Samples

A total of 150 breast tumor DNA samples were available for analysis, comprising of three groups: germline *BRCA1* mutated tumors [henceforth referred to as *BRCA1* (n=47)]*,* germline *BRCA1* wild type tumors from women from high risk families [BRCAx (n=65)], and the designated test variant tumors (n=38). Thirty-seven of these samples (14 *BRCA1*, 23 BRCAx) were previously extracted and described.^13^ Ninety samples of known *BRCA1/2* germline status [BRCAx (n=43), *BRCA1* (n=32), *BRCA1* test variants (n=15)] were collected by the Kathleen Cunningham Foundation for Research into Breast Cancer (kConFab) consortium. Ethical approval for recruitment was obtained from the institutional review boards or ethic committees of all sites, and written informed consent was given by each participant.^34^ The tumors designated as BRCAx came from women from high-risk families ascertained by kConFab and, in each case, the tumor donor had undergone germline *BRCA1/2* mutation testing by full sequencing of the coding region and splice junctions and multiplex ligation dependent probe amplification (http://www.kconfab.org/). Additional tumors from *BRCA1* test variant carriers were collected by the AFFECT study (n=23), for which ethics approval for recruitment was obtained from Brighton East Ethics Committee (REC: 06/Q1907/135) and each participant gave written informed consent. Although test variants were considered unclassified at study initiation, additional information has since allowed class to be assigned to at least some of these (see below). All variants are described using the cDNA nucleotide numbering which uses +1 as the A of the ATG translation initiation codon in the reference sequence, with the initiation codon as codon 1. A representative section of each FFPE tumor sample was stained by hematoxylin and eosin staining and evaluated by a pathologist to verify tumor content (>70% tumor) and histology; between 3 and 9 unstained slides from each tumor were needle-macro dissected, before standard phenol/chloroform DNA extraction and ethanol precipitation. The locations of the exonic sequence variants (pathogenic and test variants) assessed within the *BRCA1* gene are represented in **Figure 1A** (6 additional intronic variations investigated are not shown). Several variants had tumor samples from more than one carrier: seven of the designated test set (**Table 3**) and nine of the pathogenic variant set (**Table S3**). This study was approved by South West London REC4 (REC: 11/LO/0145).

### Laboratory analyses

#### Analysis of candidate regions

Candidate regions were identified previously^13^ and, in addition, using likelihood ratio analysis on publicly available Illumina Goldengate array data for *BRCA1* and BRCAx breast cancer tumors.^14^ All pyrosequencing assays were designed using the PyroMark Assay Design software. A common tag was placed on either the forward or reverse primer (depending on the strand to be sequenced) and a common universal biotinylated primer was used for all reactions in a semi-nested two round PCR assay.^35^ PCR primers, cycling conditions, sequencing primers, and sequence to analyze are detailed in **Table S5**. Assays were optimized with fully methylated gDNA (100%) (Zymo Research) compared to unmethylated DNA (0%, whole genome amplified DNA (Genomiphi V2, GE Healthcare). All PCR products were confirmed to be single bands by agarose gel electrophoresis. Assay quality was further assessed by comparing matched FFPE and fresh frozen samples with correlation coefficients ranging from R^2^=0.65 to 0.83 and agreement between array and pyrosequencing measured using intraclass correlation (Table1). Methylation values were calculated as an average of all CpG sites within each assay, as determined by the Pyro-QCpG software (Qiagen).

#### Illumina 450K BeadChip genome-wide analysis

Array sample selection was based upon DNA concentration quantified by Picogreen fluorescent nucleic acid stain (Invitrogen), qPCR quality control performance (Illumina FFPE QC kit) and multiplex GAPDH PCR.^36^ Bisulphite conversion of 500 ng of each sample was performed using the EZ-96 DNA Methylation-Gold™ Kit according to the manufacturer's protocol (Zymo Research, Orange, CA). Samples underwent restoration using the Illumina Infinium HD Restoration protocol, and 4 μl of bisulphite-converted restored DNA was used for hybridization on the Infinium HumanMethylation450 BeadChip, following the Illumina Infinium HD Methylation protocol. Hybridization, scanning, and raw data processing was performed by UCL Genomics (www.genomics.ucl.ac.uk/). The intensities of the images were extracted using the GenomeStudio (v.2011.1) Methylation module (1.9.0) software, which normalizes within-sample data using different internal controls that are present on the HumanMethylation450 BeadChip and internal background probes. The methylation score for each CpG was represented as a β value according to the fluorescent intensity ratio representing any value between 0 (unmethylated) and 1 (completely methylated). Raw microarray data and processed normalized data will be available from Gene Expression Omnibus (GEO) (GSE72277).  Post-array sample QC was implemented to check technical aspects of the array (staining, hybridization, target removal, extension, bisulphite conversion, specificity, non-polymorphism, and negative controls) using a threshold of >3SD outside the normal distribution of each probe set across all samples. In total, 54 samples [90%: *BRCA1* (n=18), BRCAx (n=19), *BRCA1* test variant (n=17)] passed these assessments. Detection *P*-values were also used to evaluate probe performance, and probes that failed in more than 20% of samples were discarded, leaving 482351/485577 probes (99.3%) available for analysis. Peak-based correction was used to normalize the data between the two probe types,^23^ and COMBAT^37^ was used to correct for batch effect between chips.

### Statistical analysis

Wilcoxon rank sum test was used to determine statistical significance between methylation data (pyrosequencing percentage or 450K array β value) of the *BRCA1* and BRCAx group, with FDR adjusted *P*<0.05 considered significant. A generalized linear model (glm), implemented in R, was used to interrogate the independence of mutation status, ER status, and grade, and to generate a prediction model, which was utilized by the predict command in R for predicting variant pathogenicity. The clustcomp command from the clusterCons R package was used to perform consensus clustering on β value of the 18 probes of all samples on the Illumina 450K BeadChip array. Pearson's correlation coefficient between probes was calculated using the cor function in R, and reported as an R^2^ value.

A generalized linear model was constructed and used to predict pathogenicity using the “predict” function in R version 2.15.1. The resulting probabilities were converted to likelihood ratios by calculating probability/1-probability. The posterior probability was calculated as follows: the posterior odds was calculated using prior probabilities obtained from sequence bioinformatics (as described below) multiplied by the likelihood ratio of each variant and 1/1-prior probability. This was then converted to a poster probability by dividing this value by itself plus 1.

### Assessment of test set variant classifications based on current evidence

After completion of analysis and during manuscript preparation, an extensive literature review and database search was undertaken to identify the most up-to-date information pertinent to variant classification for the entire test set variants. The most up-to-date posterior probability based on multifactorial likelihood analysis was accessed from a public website displaying information collated from the literature (http://brca.iarc.fr/LOVD/home.php). For variants that did not reach Class 1 (non pathogenic or of little clinical significance) or Class 5 (pathogenic), the posterior probability was recalculated using additional information relevant to multifactorial analysis. Methods used were as described previously,^38^ with two exceptions. First, prior probability of pathogenicity was updated to incorporate possible effects of sequence variation of splicing, based on *in silico* splicing prediction algorithms adapted for this purpose (Tavtigian, personal communication). Specifically, all exonic sequence variants plus intronic variants detected in the vicinity of the splice junction sequences with allele frequencies <0.5% were scored for their potential impact on splicing using MaxEntScan (MES), which computes the maximum entropy score of a given sequence using splice site models trained on human data.^39^ MES was calibrated by calculating the average and standard deviation of MES scores for the wild type splice junctions in *BRCA1*, *BRCA2*, and *ATM*, allowing raw MES scores to be converted into z-scores. Based on *BRCA1* and *BRCA2* mutation screening data used previously to calibrate Align-GVGD,^7,9^ rare variants that fall within the acceptor or donor region and reduce the MES score for the splice signal in which they fall showed ~97% probability to damage splice junction function when they result in a calibrated MES z-score <-2 (for donors) or  <-1.5 (for acceptors), or ~34% probability, when they result in a calibrated MES z-score between -2 and 0 (for donors) or between -1.5 and 0.5 (for acceptors). Additionally, exonic rare variants that increased the MES donor score of their sequence context and resulted in a calibrated MES donor z-score >0 had ~64% probability to create a *de novo* donor, while if they resulted in a calibrated MES z-score between -2 and 0, the probability to create a *de novo* donor was ~30% (Spurdle, Goldgar, Parsons, unpublished data). These MES-based rules were used to identify rare sequence variants that are likely to alter mRNA splicing. For exonic variants that resulted in a missense substitution, the higher of the two priors (missense vs. splicing) took precedence for multifactorial likelihood analysis. Secondly, revised pathology LRs were drawn from a recent large-scale age-stratified analysis of 4477 *BRCA1* mutation carriers, 2565 *BRCA2* mutation carriers, and 47565 breast cancer cases with no known mutation (Spurdle et al., 2015).  Additional information included breast tumor ER and grade status extracted from the literature, family history, segregation and co-occurrence likelihood ratios (LRs), assessed from a previously described data set,^7^ and further segregation and pathology data, compiled for the variants identified through the kConFab and AFFECT studies. Published mRNA assay data were identified from the literature, where this was available. Current class, and rationale for classification, is summarized in **Table S4** for all test variants.

## Authors' Contributions

Experiments and analysis were performed by KJF. KJF and NSS participated in sample preparation. M-EB performed pathological review of all tumors. ABS, MP, JM, and DEG provided samples and/or participated in data analysis and interpretation. JMF, RB, ABS, and JM conceived the study design and coordination. KJF and JMF drafted the manuscript.

## Declarations

Ethical approval references are listed in the methods. All authors read and approved the final manuscript.
