# Supplementary material for: DNA methylation profiling to assess pathogenicity of BRCA1 unclassified variants in breast cancer
Source: Epigenetics. 2016 Jan 4;10(12):1121–32. doi: 10.1080/15592294.2015.1111504 (PMC4844213; doi:10.1080/15592294.2015.1111504)
Supplement: Supplemental_.zip [file kepi-10-12-1111504-s001.zip › Supplemental Figure keys.docx]

Supp. Table S1: Prior candidate gene validation by pyrosequencing

Supp. Table S2: Individual sample predictions for *BRCA1* test variant samples analyzed

Supp. Table S3: Individual sample and combined summary predictions for *BRCA1* and BRCAx samples analyzed.

Supp. Table S4: Current class and rationale for classification of test variants

Supp. Table S5: PCR primers, annealing temperatures, sequencing primer, and sequence to analyze used in pyrosequencing assay
