# Supplementary material for: DNA methylation profiling to assess pathogenicity of BRCA1 unclassified variants in breast cancer
Source: Epigenetics. 2016 Jan 4;10(12):1121–32. doi: 10.1080/15592294.2015.1111504 (PMC4844213; doi:10.1080/15592294.2015.1111504)
Supplement: Supplemental_.zip [file kepi-10-12-1111504-s001.zip › 2015EPI0283R-s01.pdf]

Supp. Table S1: Prior candidate gene validation by pyrosequencing

Pyro Region	BRCA1 total n=	BRCAx total n=	BRCA UV total n=	methylation % (pyrosequencing)		wilcox p value BRCA1 vs BRCAx	Logistic regression p values				
									meth~mut+ER		
				Median methylation BRCA1	Median methylation BRCAx		meth~mut	meth~ER	Mut P.val	ER P.val	meth ~mut + ER + mut*ER interaction
**CD9**	44	60	37	12.77	34.09	1.25E-07	1.71E-03	5.29E-04	0.10	3.34E-02	0.95
**CD40**	42	62	35	46.76	35.17	0.02	0.32	0.95	0.30	0.57	0.51
**SGK1**	39	61	31	21.90	43.20	3.36E-07	3.16E-04	2.25E-05	0.10	2.96E-03	0.20
**FGF2-S2**	35	41	28	8.60	26.68	8.08E-05	3.33E-04	9.21E-05	0.06	1.81E-02	0.21
**FGF2-S3**	40	44	32	36.63	58.35	1.83E-03	1.01E-03	1.41E-04	0.08	1.08E-02	0.65
**ERCC3**	35	42	33	47.99	85.04	4.22E-08	4.24E-05	1.61E-05	0.23	1.82E-04	0.99

Pyro region	BRCA1 total n=	BRCAx total n=	BRCA UV total n=	methylation % (pyrosequencing)		wilcox p value BRCA1 vs BRCAx	chi squared BRCA1 vs BRCAx p value
				Median methylation BRCA1	Median methylation BRCAx		
**DES**	32	34	27	7.96	10.29	0.79	0.63
**DLC1**	46	60	37	30.61	25.89	0.24	0.35
**MEST**	46	66	38	32.98	25.13	0.09	0.11
**PCDH1**	43	50	38	3.83	3.72	0.21	0.14
**PKD2**	41	49	36	1.39	1.51	0.48	0.61
**BRCA1**	38	49	31	4.34	4.85	0.11	0.58
