# Supplementary material for: DNA methylation profiling to assess pathogenicity of BRCA1 unclassified variants in breast cancer
Source: Epigenetics. 2016 Jan 4;10(12):1121–32. doi: 10.1080/15592294.2015.1111504 (PMC4844213; doi:10.1080/15592294.2015.1111504)
Supplement: Supplemental_.zip [file kepi-10-12-1111504-s001.zip › 2015EPI0283R-s02.pdf]

Supp. Table S2: Individual sample predictions for BRCA1 test variant samples analysed.

StudyID	HGVS Nuc	HGVS Prot	Variant	Independent tumour sample (Y/N)	data available per sample (BACH2 meth/C8orf31 meth/LOC meth/ER status/grade *	ER status (1=postive, 0=negative)	grade	Probability meth only model	LR meth only model	On 450K array?
BRCA1.UV.1	c.4103C>T	Ala1368Val	A1368V	N	11111	1	1	0.1630	0.1948	YES
BRCA1.UV.2	c.4103C>T	Ala1368Val	A1368V	N	11111	1	1	0.3316	0.4961	NO
BRCA1.UV.3	c.641A>G	Asp214Gly	D214G	Y	01111	1	3	0.0308	0.0317	YES
BRCA1.UV.4	c.823G>A	Gly275Ser	G275S	N	11011	1	2	0.2322	0.3024	NO
BRCA1.UV.5	c.823G>A	Gly275Ser	G275S	N	11011	1	2	0.0900	0.0989	NO
BRCA1.UV.6	c.1984_1992del	H662-R664del	H662-R664del	Y	11111	0	3	0.7173	2.5369	YES
BRCA1.UV.7	c.2912A>G	His971Arg	H971R	Y	11111	1	1	0.6520	1.8732	NO
BRCA1.UV.8	c.203T>G	Ile68Arg	I68R	Y	11000	NA	NA	0.0063	0.0063	NO
BRCA1.UV.9	c.593+16C>G	IVS	BRCA1 IVS 9+16C>G	Y	11111 †	0	3	0.8908	8.1575	YES
BRCA1.UV.10	c.4185+9C>T	IVS	BRCA1 IVS 12+9 C>T	Y	01111	1	2	0.1125	0.1267	YES
BRCA1.UV.11	c.4484+2_4484+3ins8	IVS	BRCA1 IVS 14+2 ins 8	y	11110	0	NA	0.5883	1.4288	YES
BRCA1.UV.12	c.4485-8C>T	IVS	BRCA1 IVS 14-8 C>T	Y	11111	1	3	0.1763	0.2140	YES
BRCA1.UV.13	c.5194-12G>A	IVS	BRCA1 IVS 19-12 G>A	Y	11111	0	3	0.9646	27.2475	YES
BRCA1.UV.14	c.5194-12G>A	IVS	BRCA1 IVS 19-12 G>A	Y	01111	1	3	0.9515	19.6316	YES
BRCA1.UV.15	c.5467+5G>C	IVS	BRCA1 IVS 23+5 G>C	Y	11111	1	3	0.2256	0.2913	NO
BRCA1.UV.16	c.454C>T	Leu152Phe	L152F	Y	01100	NA	NA	0.0076	0.0076	YES
BRCA1.UV.17	c.4991T>C	Leu1664Pro	L1664P	Y	11010	0	NA	0.9029	9.2943	YES
BRCA1.UV.18	c.1534C>T	Leu512Phe	L512F	Y	11001	NA	1	0.9518	19.7525	NO
BRCA1.UV.19	c.4955T>A	Met1652Lys	M1652K	Y	11000	NA	NA	0.9374	14.9706	YES
BRCA1.UV.20	c.3708T>G	Asn1236Lys	N1236K	Y	01111	0	3	0.4504	0.8194	YES
BRCA1.UV.21	c.1036C>T	Pro346Ser	P346S	Y	01111	1	1	0.0219	0.0224	YES
BRCA1.UV.22	c.2180C>T	Pro727Leu	P727L	Y	11111	1	1	0.1476	0.1731	YES
BRCA1.UV.23	c.4039A>G	Arg1347Gly	R1347G	Y	01001	NA	3	0.8200	4.5566	YES
BRCA1.UV.24	c.4039A>G	Arg1347Gly	R1347G	Y	11000	NA	NA	0.0950	0.1050	NO
BRCA1.UV.25	c.5096G>A	Arg1699Gln	R1699Q	Y	11000	NA	NA	0.9619	25.2180	NO
BRCA1.UV.26	c.5284A>G	Arg1762Gly	R1762G	Y	11100	NA	NA	0.0783	0.0850	NO
BRCA1.UV.27	c.1486C>T	Arg496Cys	R496C	Y	11101	NA	2	0.9158	10.8764	YES
BRCA1.UV.28	c.1486C>T	Arg496Cys	R496C	Y	11001	NA	1	0.0125	0.0126	NO
BRCA1.UV.29	c.1486C>T	Arg496Cys	R496C	Y	11100	NA	NA	0.0301	0.0310	YES
BRCA1.UV.30	c.2521C>T	Arg841Trp	R841W	N	11000	NA	NA	0.1113	0.1253	NO
BRCA1.UV.31	c.2521C>T	Arg841Trp	R841W	N	11000	NA	NA	0.4891	0.9574	NO
BRCA1.UV.32	c.2521C>T	Arg841Trp	R841W	Y	01000	NA	NA	0.0227	0.0232	NO
BRCA1.UV.33	c.4963T>C	Ser 1655Pro	S1655P	Y	11100	NA	NA	0.9569	22.1870	YES
BRCA1.UV.34	c.4963T>C	Ser 1655Pro	S1655P	Y	11011	1	3	0.4201	0.7245	NO
BRCA1.UV.35	c.4963T>C	Ser 1655Pro	S1655P	Y	11000	NA	NA	0.5684	1.3168	NO
BRCA1.UV.36	c.551C>T	Ser184Phe	S184F	Y	11111	1	3	0.3628	0.5695	YES
BRCA1.UV.37	c.1423A>T	Ser475Cys	S475C	Y	11001	NA	3	0.5138	1.0568	NO

* representation of the data available for each sample. 1 indicates the methylation for the specified loci/ER status/grade is present, 0 indicates it was missing.
† BRCA1 promoter methylated tumour
