# Supplementary material for: DNA methylation profiling to assess pathogenicity of BRCA1 unclassified variants in breast cancer
Source: Epigenetics. 2016 Jan 4;10(12):1121–32. doi: 10.1080/15592294.2015.1111504 (PMC4844213; doi:10.1080/15592294.2015.1111504)
Supplement: Supplemental_.zip [file kepi-10-12-1111504-s001.zip › 2015EPI0283R-s03.pdf]

Supp. Table S3: Individual sample and combined summary predictions for BRCA1 and BRCAx samples analysed.

StudyID	Mutation	HGVS Nuc	HGVS Prot	variant	Mutation status	probability meth only	LR from meth only model	log LR meth	ER Status cat	Grade	On 450k array?
BRCA1.path.1	BRCA1	c.5467 G>A	p.Ala1823Thr	A1823T	BRCA1	0.9672	29.4964	1.4698	0	3	NO
BRCA1.path.2	BRCA1	c.131 G>T	p.Cys44Phe	C44F	BRCA1	0.9355	14.5070	1.1616	0	3	YES
BRCA1.path.3	BRCA1	c.181 T>G	p.Cys61Gly	C61G	BRCA1	0.9924	131.1372	2.1177	0	3	NO
BRCA1.path.4	BRCA1	c.181 T>G	p.Cys61Gly	C61G	BRCA1	0.6897	2.2224	0.3468	0	2	NO
BRCA1.path.5	BRCA1	c.181 T>G	p.Cys61Gly	C61G	BRCA1	0.9727	35.6047	1.5515			NO
BRCA1.path.6	BRCA1	c.181 T>G	p.Cys61Gly	C61G	BRCA1	0.9255	12.4155	1.0940	0	3	NO
BRCA1.path.7	BRCA1	c.181 T>G	p.Cys61Gly	C61G	BRCA1	0.9612	24.7797	1.3941	0	3	YES
BRCA1.path.8	BRCA1	c.181 T>G	p.Cys61Gly	C61G	BRCA1	0.8697	6.6768	0.8246	1	3	YES
BRCA1.path.9	BRCA1	c.181 T>G	p.Cys61Gly	C61G	BRCA1	0.3433	0.5227	-0.2818	1	3	YES
BRCA1.path.10	BRCA1	c.181 T>G	p.Cys61Gly	C61G	BRCA1	0.6725	2.0531	0.3124	0	3	YES
BRCA1.path.11	BRCA1	c.5212 G>A	p.Gly1738Arg	G1738R	BRCA1	0.7529	3.0461	0.4837	1	2	YES
BRCA1.path.12	BRCA1	c.5212 G>A	p.Gly1738Arg	G1738R	BRCA1	0.6365	1.7507	0.2432	0	3	YES
BRCA1.path.13	BRCA1	c.5324 T>A	p.Met1775Lys	M1775K	BRCA1	0.7415	2.8688	0.4577	0	3	YES
BRCA1.path.14	BRCA1	c.3256_3257 ins GA	p.Leu1086X	STOP 1087	BRCA1	0.9790	46.6477	1.6688	0	3	YES
BRCA1.path.15	BRCA1	c.3256_3257 ins GA	p.Leu1086X	STOP 1087	BRCA1	0.9840	61.3503	1.7878	1		YES
BRCA1.path.16	BRCA1	c.3700_3704 del GTAAA	p.Val1234GlnfsX8	STOP 1242	BRCA1	0.9973	371.0363	2.5694	0	3	NO
BRCA1.path.17	BRCA1	c.4936 del G	p.Val1646SerfsX12	STOP 1657	BRCA1	0.4908	0.9637	-0.0161	0	3	NO
BRCA1.path.18	BRCA1	c.4936 del G	p.Val1646SerfsX12	STOP 1657	BRCA1	0.9667	28.9989	1.4624	0	3	YES
BRCA1.path.19	BRCA1	c.5333-36_5406+400del510		STOP 1805	BRCA1	0.9792	47.0108	1.6722			NO
BRCA1.path.20	BRCA1	c.5503 C>T	p.Arg1835X	STOP 1835	BRCA1	0.2086	0.2636	-0.5790	1	2	NO
BRCA1.path.21	BRCA1	c.5503 C>T	p.Arg1835X	STOP 1835	BRCA1	0.9955	220.5438	2.3435	0	3	YES
BRCA1.path.22	BRCA1	c.1018 del G	p.Val340X	STOP 340	BRCA1	0.9884	84.8635	1.9287	0	3	YES
BRCA1.path.23	BRCA1	c.70_80 del TGTCCCATCTG	p.Cys24SerfsX13	STOP 36	BRCA1	0.9773	43.0440	1.6339	0		YES
BRCA1.path.24	BRCA1	c.70_80 del TGTCCCATCTG	p.Cys24SerfsX13	STOP 36	BRCA1	0.9616	25.0301	1.3985	0	3	YES
BRCA1.path.25	BRCA1	c.2071 del A	p.Arg691AspfsX10	STOP 700	BRCA1	0.9410	15.9381	1.2024	0	3	NO
BRCA1.path.26	BRCA1	c.2071 del A	p.Arg691AspfsX10	STOP 700	BRCA1	0.8603	6.1598	0.7896	0	3	YES
BRCA1.path.27	BRCA1	c.2071 del A	p.Arg691AspfsX10	STOP 700	BRCA1	0.8380	5.1710	0.7136	0	3	YES
BRCA1.path.28	BRCA1	c.2071 del A	p.Arg691AspfsX10	STOP 700	BRCA1	0.9779	44.2523	1.6459		3	YES
BRCA1.path.29	BRCA1	c.2681_2682 del AA	p.Lys894ThrfsX8	STOP 901	BRCA1	0.9419	16.2219	1.2101	1	2	NO
BRCA1.path.30	BRCA1	c.2681_2682 del AA	p.Lys894ThrfsX8	STOP 901	BRCA1	0.8963	8.6420	0.9366	1		YES
BRCA1.path.31	BRCA1	unknown		unknown	BRCA1	0.9718	34.4197	1.5368		3	NO
BRCA1.path.32	BRCA1	unknown		unknown	BRCA1	0.4012	0.6699	-0.1740	0	3	NO
BRCA1.path.33	BRCA1	unknown		unknown	BRCA1	0.6108	1.5693	0.1957	1	2	NO
BRCA1.path.34	BRCA1	unknown		unknown	BRCA1	0.9573	22.4036	1.3503	1	3	NO
BRCA1.path.35	BRCA1	unknown		unknown	BRCA1	0.0451	0.0473	-1.3254	0	3	NO
BRCA1.path.36	BRCA1	unknown		unknown	BRCA1	0.0925	0.1019	-0.9918	0	2	NO
BRCA1.path.37	BRCA1	unknown		unknown	BRCA1	0.0532	0.0562	-1.2503	1	3	NO
BRCA1.path.38	BRCA1	unknown		unknown	BRCA1	0.3761	0.6027	-0.2199	0	3	NO
BRCA1.path.39	BRCA1	unknown		unknown	BRCA1	0.0486	0.0511	-1.2913	0	3	NO
BRCA1.path.40	BRCA1	unknown		unknown	BRCA1	0.2930	0.4143	-0.3826	0	3	NO
BRCA1.path.41	BRCA1	c.5513 T>A	p.Val1838Glu	V1838E	BRCA1	0.7697	3.3425	0.5241		3	NO
BRCA1.path.42	BRCA1	c.5513 T>A	p.Val1838Glu	V1838E	BRCA1	0.9782	44.9261	1.6525		2	YES
BRCAX.1	BRCAx	x		x	BRCAX	0.0631	0.0673	-1.1717	1	3	NO
BRCAX.2	BRCAx	x		x	BRCAX	0.0039	0.0039	-2.4110	1	1	NO

BRCAX.3	BRCAx	x		x	BRCAX	0.4849	0.9412	-0.0263	1	1	NO
BRCAX.4	BRCAx	x		x	BRCAX	0.6374	1.7578	0.2450		1	NO
BRCAX.5	BRCAx	x		x	BRCAX	0.6701	2.0316	0.3078			YES
BRCAX.6	BRCAx	x		x	BRCAX	0.4508	0.8207	-0.0858	1		NO
BRCAX.7	BRCAx	x		x	BRCAX	0.0136	0.0138	-1.8607	0	3	NO
BRCAX.8	BRCAx	x		x	BRCAX	0.0356	0.0369	-1.4329		2	NO
BRCAX.9	BRCAx	x		x	BRCAX	0.4651	0.8694	-0.0608		2	NO
BRCAX.10	BRCAx	x		x	BRCAX	0.0237	0.0242	-1.6156	1	2	NO
BRCAX.11	BRCAx	x		x	BRCAX	0.6620	1.9589	0.2920	1	2	NO
BRCAX.12	BRCAx	x		x	BRCAX	0.0961	0.1063	-0.9736	1	2	NO
BRCAX.13	BRCAx	x		x	BRCAX	0.2377	0.3118	-0.5062		1	NO
BRCAX.14	BRCAx	x		x	BRCAX	0.0938	0.1035	-0.9849			NO
BRCAX.15	BRCAx	x		x	BRCAX	0.0692	0.0743	-1.1290	1	1	NO
BRCAX.16	BRCAx	x		x	BRCAX	0.3883	0.6348	-0.1974	1	2	NO
BRCAX.17	BRCAx	x		x	BRCAX	0.0546	0.0578	-1.2384	1	3	NO
BRCAX.18	BRCAx	x		x	BRCAX	0.0108	0.0110	-1.9606			NO
BRCAX.19	BRCAx	x		x	BRCAX	0.0934	0.1031	-0.9869	1	2	NO
BRCAX.20	BRCAx	x		x	BRCAX	0.2723	0.3742	-0.4269	1	3	NO
BRCAX.21	BRCAx	x		x	BRCAX	0.2125	0.2698	-0.5689	1	3	NO
BRCAX.22	BRCAx	x		x	BRCAX	0.2256	0.2913	-0.5356	0	3	NO
BRCAX.23	BRCAx	x		x	BRCAX	0.4951	0.9807	-0.0084	1	2	NO
BRCAX.24	BRCAx	x		x	BRCAX	0.0432	0.0451	-1.3457	1	2	NO
BRCAX.25	BRCAx	x		x	BRCAX	0.2813	0.3914	-0.4074	1	3	NO
BRCAX.26	BRCAx	x		x	BRCAX	0.0361	0.0375	-1.4261	1	3	NO
BRCAX.27	BRCAx	x		x	BRCAX	0.0506	0.0533	-1.2730	1	3	NO
BRCAX.28	BRCAx	x		x	BRCAX	0.0177	0.0180	-1.7452	1	2	NO
BRCAX.29	BRCAx	x		x	BRCAX	0.0108	0.0109	-1.9638	1	2	NO
BRCAX.30	BRCAx	x		x	BRCAX	0.9567	22.1016	1.3444	1	3	NO
BRCAX.31	BRCAx	x		x	BRCAX	0.4963	0.9853	-0.0064	1		NO
BRCAX.32	BRCAx	x		x	BRCAX	0.3813	0.6164	-0.2101			NO
BRCAX.33	BRCAx	x		x	BRCAX	0.1139	0.1286	-0.8908	1	1	NO
BRCAX.34	BRCAx	x		x	BRCAX	0.2135	0.2715	-0.5662	0	3	NO
BRCAX.35	BRCAx	x		x	BRCAX	0.1701	0.2050	-0.6883		2	NO
BRCAX.36	BRCAx	x		x	BRCAX	0.5827	1.3963	0.1450		1	NO
BRCAX.37	BRCAx	x		x	BRCAX	0.0030	0.0030	-2.5273	1	1	YES
BRCAX.38	BRCAx	x		x	BRCAX	0.1987	0.2479	-0.6057	0	2	YES
BRCAX.39	BRCAx	x		x	BRCAX	0.0155	0.0157	-1.8041	1	2	YES
BRCAX.40	BRCAx	x		x	BRCAX	0.2047	0.2574	-0.5894		3	YES
BRCAX.41	BRCAx	x		x	BRCAX	0.2953	0.4191	-0.3777	1	2	YES
BRCAX.42	BRCAx	x		x	BRCAX	0.4775	0.9138	-0.0391	1	2	YES
BRCAX.43	BRCAx	x		x	BRCAX	0.6972	2.3026	0.3622	1	2	YES
BRCAX.44	BRCAx	x		x	BRCAX	0.1624	0.1939	-0.7124	1	2	YES
BRCAX.45	BRCAx	x		x	BRCAX	0.2492	0.3319	-0.4790	1	2	YES
BRCAX.46	BRCAx	x		x	BRCAX	0.1311	0.1509	-0.8213	1	1	YES
BRCAX.47	BRCAx **&**	x		x	BRCAX	0.8778	7.1832	0.8563	0	3	YES
BRCAX.48	BRCAx	x		x	BRCAX	0.0421	0.0440	-1.3568		2	YES
BRCAX.49	BRCAx	x		x	BRCAX	0.3191	0.4687	-0.3291		2	YES
BRCAX.50	BRCAx	x		x	BRCAX	0.4035	0.6765	-0.1697	0	3	YES
BRCAX.51	BRCAx	x		x	BRCAX	0.1065	0.1192	-0.9237	1	2	YES
BRCAX.52	BRCAx	x		x	BRCAX	0.0897	0.0986	-1.0062	1	2	YES

BRCAX.53	BRCAx	x		x	BRCAX	0.7243	2.6270	0.4195	1	3	YES
BRCAX.54	BRCAx	x		x	BRCAX	0.1582	0.1879	-0.7260	1	3	YES
BRCAX.55	BRCAx	x		x	BRCAX	0.7390	2.8319	0.4521	0	3	YES

& BRCA1 promoter methylated tumour

**Number of tumours with variant**	**HGVS Nuc**	**HGVS Prot**	**Protein Change**	**Combined LR: Logistic Regression Model**
8	c.181 T>G	p.Cys61Gly	C61G	22872971.23
4	c.2071 del A	p.Arg691AspfsX10	STOP 700	22465.67004
2	c.5212 G>A	p.Gly1738Arg	G1738R	5.332763131
2	c.3256_3257 ins GA	p.Leu1086X	STOP 1087	2861.85276
2	c.4936 del G	p.Val1646SerfsX12	STOP 1657	27.94630391
2	c.5503 C>T	p.Arg1835X	STOP 1835	58.13796852
2	c.70_80 del TGTCCCATCTG	p.Cys24SerfsX13	STOP 36	1077.397332
2	c.2681_2682 del CT	p.Lys894ThrfsX8	STOP 901	140.1901393
2	c.5513 T>A	p.Val1838Glu	V1838E	150.163233
1	c.5467 G>A	p.Ala1823Thr	A1823T	29.4964
1	c.131 G>T	p.Cys44Phe	C44F	14.5070
1	c.5324 T>A	p.Met1775Lys	M1775K	2.8688
1	c.3700_3704 del GTAAA	p.Val1234GlnfsX8	STOP 1242	371.0363
1	c.5333-36_5406+400del510		STOP 1805	47.0108
1	c.1018 del G	p.Val340X	STOP 340	84.8635
