# Supplementary material for: DNA methylation profiling to assess pathogenicity of BRCA1 unclassified variants in breast cancer
Source: Epigenetics. 2016 Jan 4;10(12):1121–32. doi: 10.1080/15592294.2015.1111504 (PMC4844213; doi:10.1080/15592294.2015.1111504)
Supplement: Supplemental_.zip [file kepi-10-12-1111504-s001.zip › 2015EPI0283R-s04.pdf]

Supp. Table S4: Current class and rationale for classification of test variants.

Gene	HGVS Nucleotide	HGVS Protein	BIC Nomenclature	Data Source(s) for Statistical Analysis*	Prior Probability of Pathogenicity**	Segregation LR	Pathology LR	Co-occurrence LR	Family History LR	Combined LR	Posterior Probability of Pathogenicity	IARC Class***	Class Description
BRCA1	c.1036C>T	p.(Pro346Ser)	P346S	This paper	0.02	1	0.21	1	1	0.21	0.004267425	2	Likely not pathogenic
BRCA1	c.1423A>T	p.(Ser475Cys)	S475C	This paper	0.02	0.1944	1	1	1	0.1944	0.003951669	2	Likely not pathogenic
BRCA1	c.1486C>T	p.(Arg496Cys)	R496C	Ref [1] (Primary data). Ref [2] (Posterior probability calculation).	0.02	1.95	1	0.0224	1	0.04368	0.000890635	1	Not pathogenic
BRCA1	c.1534C>T	p.(Leu512Phe)	L512F	This paper	0.02	0.9999	0.12	1.73925	0.03357	0.00700569	0.000142953	1	Not pathogenic
BRCA1	c.1984_1992del	p.(His662_Arg664del)	H662-R664del	This paper	0.02	1	3.16	1	1	3.16	0.060582822	3	Uncertain
BRCA1	c.203T>G ¥	p.(Ile68Arg)	I68R	This paper - no additional clinical data	0.66							3	Uncertain
BRCA1	c.2180C>T	p.(Pro727Leu)	P727L	This paper	0.02	0.0628	0.13	1	1	0.008164	0.000166584	1	Not pathogenic
BRCA1	c.2521C>T	p.(Arg841Trp)	R841W	Ref [3] (Primary data). Ref [2] (Posterior probability calculation).	0.02	0.000000004	1	0.028	1	1.12E-10	2.28571E-12	1	Not pathogenic
BRCA1	c.2912A>G	p.(His971Arg)	H971R	This paper	0.02	1.937	0.0064	1	1	0.0123968	0.000252932	1	Not pathogenic
BRCA1	c.3708T>G ¥	p.(Asn1236Lys)	N1236K	This paper	0.02	0.0556	1.0744	11.465	1	0.68488058		3	Uncertain
BRCA1	c.3848A>G	p.(His1283Arg)	H1283R	This paper	0.02	1	1	1.13908	0.172823	0.19685968	0.004001468	2	Likely not pathogenic
BRCA1	c.4039A>G	p.(Arg1347Gly)	R1347G	Ref [4] (Primary data).Ref [2] (Posterior probability calculation).	0.02	1	1	1E-10	1	1E-10	2.04082E-12	1	Not pathogenic
BRCA1	c.4103C>T	p.(Ala1368Val)	A1368V	This paper	0.02	1	0.08	1	1	0.08	0.001629992	2	Likely not pathogenic
BRCA1	c.4185+9C>T		BRCA1 IVS 12+9 C>T	This paper	0.02	0.1998	0.57	1	1	0.113886	0.002318815	2	Likely not pathogenic
BRCA1	c.4479_4484+2dup ^		BRCA1 IVS 14+2 ins 8	This paper	0.97	0.9611	3.16	1	1	3.037076	0.989919232	4	Likely pathogenic
BRCA1	c.4485-8C>T ¥		BRCA1 IVS 14-8 C>T	This paper	0.04	1	0.9	1	1	0.9		3	Uncertain
BRCA1	c.454C>T ¥	p.(Leu152Phe)	L152F	This paper - no additional clinical data	0.02							3	Uncertain
BRCA1	c.4955T>A ¥	p.(Met1652Lys)	M1652K	This paper	0.66	1	1	1.033092037	0.758064	0.78314939		3	Uncertain
BRCA1	c.4963T>C ¥	p.(Ser1655Pro)	S1655P	This paper	0.03	1	0.64	1	1	0.64		3	Uncertain
BRCA1	c.4991T>C	p.(Leu1664Pro)	L1664P	Ref [5] (Primary data). http://hci-exlovd.hci.utah.edu/ (Posterior probability calculation)	0.03	0.01	1	1.255952165	0.030259	0.00038003	1.17535E-05	1	Not pathogenic
BRCA1	c.5194-12G>A		BRCA1 IVS 19-12 G>A	This paper	0.34	4.613307	8.35251	1.255952165	33127.18	1603197.31	0.999998789	5	Pathogenic
BRCA1	c.5284A>G ¥	p.(Arg1762Gly)	R1762G	This paper - no additional clinical data	0.03							3	Uncertain
BRCA1	c.5467+5G>C		BRCA1 IVS 23+5 G>C	This paper	0.34	0.7125	0.64	1	1	0.456	0.190223793	3	Uncertain
BRCA1	c.551C>T ¥	p.(Ser184Phe)	S184F	This paper	0.02	1	0.64	1	1	0.64		3	Uncertain
BRCA1	c.593+16C>G ¥		BRCA1 IVS 9+16C>G	This paper	0.02	1	4.13	1	1	4.13		3	Uncertain
BRCA1	c.641A>G	p.(Asp214Gly)	D214G	This paper	0.02	4.3671	0.9	1	1	3.93039	0.074255829	3	Uncertain
BRCA1	c.823G>A	p.(Gly275Ser)	G275S	This paper	0.02	1	0.21	1	1	0.21	0.004267425	2	Likely not pathogenic
BRCA1	c.5096G>A	p.(Arg1699Gln)	R1699Q	Ref [6].									Intermediate risk

* Source is recorded as 'This paper' when multiple sources were used for primary data, including: Easton et al. 2007, AM J Hum Genet 81: 873-83; Spurdle et al. 2008, J Clin Oncol 26:1657-63; and unpublished data from kConFab.
** Prior probability of pathogenicity as per original publication where stated, or from http://priors.hci.utah.edu/PRIORS/BRCA/
*** Thresholds for defining classes as per Plon et al 2008, Hum Mutat 29: 1292-1303.
¥ Combined LR does not pass thresholds recommended as per ENIGMA BRCA classification guidelines (http://www.enigmaconsortium.org/), namely LR of <0.5 (to reach final class 2 or 1), or >2.0 (to reach final class 4 or 5). and so should be considered class 3 uncertain.
^ Previously reported as c.4484+2ins8 (Whiley et al 2011, Hum Mutat 32:678-87). Insertion is due to duplication of GGAAAGGT.

In Table References:

1: Chenevix-Trench G, Healey S, Lakhani S, Waring P, Cummings M, Brinkworth R, Deffenbaugh AM, Burbidge LA, Pruss D, Judkins T, Scholl T, Bekessy A, Marsh A, Lovelock P, Wong M, Tesoriero A, Renard H, Southey M, Hopper JL, Yannoukakos K, Brown M, Easton D, Tavtigian SV, Goldgar D, Spurdle AB; kConFab Investigators. Genetic and histopathologic evaluation of BRCA1 and BRCA2 DNA sequence variants of unknown clinical significance. Cancer Res. 2006 Feb 15;66(4):2019-27.
2: Lindor NM, Guidugli L, Wang X, Vallée MP, Monteiro AN, Tavtigian S, Goldgar DE, Couch FJ. A review of a multifactorial probability-based model for classification of BRCA1 and BRCA2 variants of uncertain significance (VUS). Hum Mutat. 2012 Jan;33(1):8-21. doi: 10.1002/humu.21627.
3: Goldgar DE, Easton DF, Deffenbaugh AM, Monteiro AN, Tavtigian SV, Couch FJ; Breast Cancer Information Core (BIC) Steering Committee. Integrated evaluation of DNA sequence variants of unknown clinical significance: application to BRCA1 and BRCA2. Am J Hum Genet. 2004 Oct;75(4):535-44.
4: Tavtigian SV, Deffenbaugh AM, Yin L, Judkins T, Scholl T, Samollow PB, de Silva D, Zharkikh A, Thomas A. Comprehensive statistical study of 452 BRCA1 missense substitutions with classification of eight recurrent substitutions as neutral. J Med Genet. 2006 Apr;43(4):295-305.
5: Whiley PJ, Parsons MT, Leary J, Tucker K, Warwick L, Dopita B, Thorne H, Lakhani SR, Goldgar DE, Brown MA, Spurdle AB. Multifactorial likelihood assessment of BRCA1 and BRCA2 missense variants confirms that BRCA1:c.122A>G(p.His41Arg) is a pathogenic mutation. PLoS One. 2014 Jan 28;9(1):e86836.
6: Spurdle AB, Whiley PJ, Thompson B, Feng B, Healey S, Brown MA, Pettigrew C; kConFab, Van Asperen CJ, Ausems MG, Kattentidt-Mouravieva AA, van den Ouweland AM; Dutch Belgium UV Consortium, Lindblom A, Pigg MH, Schmutzler RK, Engel C, Meindl A; German Consortium of Hereditary Breast and Ovarian Cancer, Caputo S, Sinilnikova OM, Lidereau R; French COVAR group collaborators, Couch FJ, Guidugli L, Hansen Tv, Thomassen M, Eccles DM, Tucker K, Benitez J, Domchek SM, Toland AE, Van Rensburg EJ, Wappenschmidt B, Borg Å, Vreeswijk MP, Goldgar DE; ENIGMA Consortium. BRCA1 R1699Q variant displaying ambiguous functional abrogation confers intermediate breast and ovarian cancer risk. J Med Genet. 2012 Aug;49(8):525-32.
