# Supplementary material for: DNA methylation profiling to assess pathogenicity of BRCA1 unclassified variants in breast cancer
Source: Epigenetics. 2016 Jan 4;10(12):1121–32. doi: 10.1080/15592294.2015.1111504 (PMC4844213; doi:10.1080/15592294.2015.1111504)
Supplement: Supplemental_.zip [file kepi-10-12-1111504-s001.zip › 2015EPI0283R-s05.pdf]

Supp. Table S5. PCR primers, annealing temperatures, sequencing primer and sequence to analyse used in pyrosequencing assay.

Gene name	RefSeqID	candidate region/probe ID	Forward primer sequence	Reverse primer sequence	Sequencing primer sequence	Sequence to analyse	annealing temperature of PCR reaction
CD9	NM_001769.3	Holm et al 2010	GGTTAAGGTTTGTTGTTAGAGTG	gacGGGACACCGCTGATCGTTTATTCCTCCTACCCCACATACTATAC	AAGGGAAGTYGTTTGGTTTA	ATTTYGTTTT YGTTTGTTTT TTTGTAAATT TTYGTTYGTT GATTATATTT TTT	50
CD40	NM_152854.2	Holm et al 2010	AGTTTTAGAGGAYGTATTGTAGAGGTAGA	gacGGGACACCGCTGATCGTTTAAAAAACCCCRCCCCTTTCCT	GAATTATAGYGAGGTGAGATTAG	GYGGTAGGAT TATTGGGYGT TYGAGYGAGG TTTTTGTTGA TTTTTTTG	50
ERCC3	NM_000122.1	Holm et al 2010	GAAGAGTTTTGGTAGAAGTATGGGTATTAA	gacGGGACACCGCTGATCGTTTA CTACCACTTACCTCCAATTTCAATA	GGGTATTAAAGGTGATTTT	GGTGAYGGTTTAGATGGAAAGGAGAAATATGTT	55
FGF2 (assay 1)	NM_002006.4	Holm et al 2010	GGGGGAGAAAGTTGAGTTTAA	gacGGGACACCGCTGATCGTTTAACTTTAACATTCCCTAAACTCC	TTTGGGGGATAAGGG	GYGGTGGAGTTTAGGGAATGTTAAAGTTT	55
FGF2 (assay 2)	NM_002006.4	Holm et al 2010	GGGGGAGAAAGTTGAGTTTAA	gacGGGACACCGCTGATCGTTTAACTTTAACATTCCCTAAACTCC	GGTTGGTTGYGTAGTAAAAG	TTTYGTAGTGTGGAGAAAGTTTAAAYGTGGTTTGGGTGGTGYGGGGGTTGGGYGGGGGTGATTTTTGGGGGATAAGGGG	55
SGK1	NM_001143676.1	Flanagan et al 2010	gacGGGACACCGCTGATCGTTTA-TTGTAGAAGGTAGGGAAGAGAGG	TCAAACTACRACTCCCAAACAT	CCCAAACATCCCCCA	ACCRCRAAATTACTAAAAAACACCRACATCRCTATTCTACAAAACCCRAACCRCCRCCTCRAACT	59
PKD2	NM_000297.3	Flanagan et al 2010	gacGGGACACCGCTGATCGTTTA-ATTTTTTTTTGGAAGGGTAGT	CACCAACAAAAAAACAATAAAATT	ACAAAAAAACAATAAAATTC	CRCRCTAAATACCRAAAATAAAACAACACTAACRAAAATAAAAAATACRCTT	50
PCDH1	NM_002587.3	Holm et al 2010	GGTTTGGGTGGGATAAGTGTAAG	gacGGGACACCGCTGATCGTTTACTCCCTCTACCATCTCTTCACTAC	GAAGTTTAGAGGAGGGT	AYGGTTTGTGTATTGGGTTTYGGGTAGTGAAGAGATGGTAGAGGGAG	55
MEST	NM_002402.3	Holm et al 2010	GGGTTTTTTTTGGGAATAGGG	gacGGGACACCGCTGATCGTTTAATAAAACCTTACCTACAAAACTCCATAT	AGAATTTTTGGTTTTAGGAAG	YGTATGYGTAATYGGTTTTTYGAAATATGGAGTTTTGTAGGTAAGG	50
DLC1	NM_182643.2	Holm et al 2010	GGGAGGTATAAGAAAGTAGAAAAGAT	gacGGGACACCGCTGATCGTTTAACTTACAACTACTTCCATCTAACATAAC	TTTTAGAGATGTGATTTAGGAA	YGTTGTTATGTTAGATGGAAGTAGTTGTAAGT	50
DES	NM_001927.3	Holm et al 2010	GGGTTTYGGTTTTAAGGGTTTT	gacGGGACACCGCTGATCGTTTAACCAATAAAAAATCCAACAACT	GTTTYGGTTTTAAGGGTTTTTTTAG	TTYGGTGAYGTTTYGYGTGTATTAGGTGTYGYGTAYGTYGGGYGGGGTYGG	50
BRCA1	NM_007294.3	Flanagan et al 2010	GTATTTTGAGAGGTTGTTGTTTAG	gacGGGACACCGCTGATCGTTTAACCCAATTATCTAAAAAACCCCACAA	GGAAAAGYGYGGGAATTATAGATAA	ATTAAAATTG YGATTGYGYG GYGTGAGTTY GTTGAGATTT TTTGGA	57
C8orf31	NM_173687.2	cg03029255	GGGAGGAGGTTGTAGGAG	gacGGGACACCGCTGATCGTTTAAAACAATCCCCAAACCTTACTA	GTTTTAATGTATTTTTTGATAT	TAYGTATGTT TAGTAAGGTT TGGGGATTG	50
1p34.2	na	cg07405182	gacGGGACACCGCTGATCGTTTATGTTAGGGGTYGGATGGTGAAT	ACTTTAATCCCCTTTCTTCTCTC	CTCTCTAACAAAACAATAACTT	TTAAAAATTC RCTACAAAAA AAAATTCACC ATCC	50
BACH2	NM_021813.2	cg24667115	gacGGGACACCGCTGATCGTTTAGGGAGGTGTTYGAGAATTAGGG	AACTAAACRTAAAAAACTACCCCTCCTT	AACTACCCCTCCTTT	AACRAAAAAA ACTATTTTAA TAAAACAACR AACRTCTTAC TTAAAATATA AAATCRCCCC ACCCTAATTC TC	50
3q25.2	na	cg02071853	gacGGGACACCGCTGATCGTTTAGTTTTGTTAGTTGTAAATGGAATATATGT	TTCCTCTTCCATAAACAAATTTTCACA	CCATAAACAAATTTTCACATC	CRATCTCTCCATCTATCAAAA	56
15q22	na	cg08036487	gacGGGACACCGCTGATCGTTTAGGTTAGGTTTAGTGGGAAGTGTT	ACCTTTATCATCATCATTATCTTTACATAT	ACTACCAATATTTTCAAATCC	TCRTTTTACTAAATAAATTAATATATATA	50
13q34	na	cg12472473	AGATGGAGGTGGTTTTATAGT	gacGGGACACCGCTGATCGTTTAATACCTCCTTTATCCCCAAATTTA	ATTTTGATTTTTTTGTTTGGT	YGAGAGTTAGYGTTTTAAATTTGGGGATA	50
8p23.1	na	cg04039177	ATTTGTAAAAGTAAGTTGTGGTATATG	gacGGGACACCGCTGATCGTTTATTAAAAACATTTACTCTAAACCAAATATA	TTGTTTAGGGTTGTGTT	AYGAGGTTATTGGTTATTTATATTTGGTT	50
LOC	NR_027238.1	cg21645762	TTGTATGAGTATGGGGTGTAGGTATG	gacGGGACACCGCTGATCGTTTAACCCTCACTCTACTCACAACTC	TTTAGGTTTAGGGAGTTT	YGYGTTYGGTTATATTTTATGTGTTGGGTATGAG	50
CRTAC1	NM_018058.6	cg23801028	TATTTTTTTGGTTTAGAGAGGTAGAAATTA	gacGGGACACCGCTGATCGTTTATCCCTCACCCCTTTAATCACAAATACTAC	GGTAGYGGGTTGGGG	GYGAGGAGAGGYGGGTAGTATTTGTGATTAAAG	50
DLC1 (450K hit)	NM_182643.2	cg00933411	gacGGGACACCGCTGATCGTTTAAAGGGAGGTATAAGAAAGTAGAAAAG	CACTTACAACTACTTCCATCTAACATA	ACTACTTCCATCTAACATAAC	AACRTTCCTAAATCACATCTCTAAAACCRCTCATAATA	50
TJAP1	NM_001146019.1	cg16759204	TTGTTAGTGGTTGTGTTTAGTTTAGA	gacGGGACACCGCTGATCGTTTACCCAAAAACTCAAATAAACCAAAACCTA	AGTTTAGAGTAGGGGT	TAGGGTTTGTYGGAAG	50
C17orf108	NM_001076680.1	cg02502358	AGGTTTAAAAAGTTTTGGAGAAGAG	gacGGGACACCGCTGATCGTTTAACCCCCCTATTCAATAATACTACTAATC	ATATTATTTATTAATAATTTAATTT	TYGTTTAGATATTAGTTGAGAGATTAGT	50
INTS1	NM_001080453.2	cg11644627	gacGGGACACCGCTGATCGTTTAGGTATTTGGAGTTTGGGTTAATTTTA	ACCCTCACCAACTAAAAAATTT	AAAAATTTTAAAAAAATCTCTATAA	AATCRTCTAAACCCRTACA	50
6q27	na	cg26074411	TTGYGGTTTTAGGTTAGTAGGTAAGT	gacGGGACACCGCTGATCGTTTACCAAAACRAAAAACCCACAATAAAATTACT	AGGTTAGTAGGTAAGTTTT	TYGTAGYGGGATTYGYGTTT	54
PLAGL1	NM_001080951.1	cg02697107	TGGGTAGATATAGGTTTATTTGGAGAT	gacGGGACACCGCTGATCGTTTAACCAAAAAAAAAAAAAATACAACCTTATAA	AGGTTTATTTGGAGATAAAGT	AYGTTTTTAT AGAATTTTTT GTTTTTTT	54
ZNF319	NM_020807.1	cg06866605	GGAGAATTTTTTGGGTTGTGT	gacGGGACACCGCTGATCGTTTAATTTAAAACCTAACTCTCCTACTACC	GTTAGATTTAGGTTTGTAGTTTTTA	TAATAYGTATTTTTGTAGGTAGTAGGAGAGTTA	57

All assays = semi nested, touchdown PCR programme with specified annealing temperature ; Universal Biotinylated Primer - BIOTAG- GGGACACCGCTGATCGTTTA
1st rd - specific F and R for each assay, 1 tagged with a universal sequence ; 2nd rd - untagged specific primer + universal biotinylated primer
